# Christiaan Barnard—The surgeon who dared: The story of the first human-to-human heart transplant

**DOI:** 10.21542/gcsp.2018.11

**Published:** 2018-06-30

**Authors:** David K.C. Cooper

**Affiliations:** Xenotransplantation Program, Department of Surgery, University of Alabama at Birmingham, Birmingham, AL, USA

## Abstract

In 2017, we celebrated the 50th anniversary of the first human heart transplant that had been carried out by the South African surgeon, Christiaan (‘Chris’) Barnard at Groote Schuur Hospital in Cape Town on December 3^rd^, 1967. The daring operation and the charismatic surgeon received immense public attention around the world. The patient’s progress was covered by the world’s media on an almost hourly basis. Although the patient, Mr. Louis Washansky, died after only 18 days, Barnard soon carried out a second transplant, and this patient led an active life for almost 19 months. Remarkably, Barnard’s fifth and sixth patients lived for almost 13 and 24 years, respectively. Barnard subsequently introduced the operation of heterotopic heart transplantation in which the donor heart acted as an auxiliary pump, with some advantages in that early era. It took great courage to carry out the first heart transplant, and this is why Barnard is remembered as a pioneer in cardiac surgery.

“It helps a man immensely to be a bit of a hero-worshipper, and the stories of the lives of the masters of medicine do much to stimulate our ambitions and rouse our sympathies.”

- Sir William Osler

In 2017, we celebrated the 50th anniversary of the first human-to-human heart transplant carried out by the South African surgeon, Christiaan (‘Chris’) Barnard (Figure 1), at Groote Schuur Hospital (GSH) in Cape Town.

## Background

Christiaan Barnard was the third son (of four) of a church minister in the rural town of Beaufort West in South Africa, approximately 300 miles inland from Cape Town. He was born on November 8th, 1922^1,2^. Although not impoverished, the family was poor (Figure 2), in part because his father dedicated himself to the spiritual care of the mixed race (‘Colored’) community in the town, and thus, in the apartheid circumstances of South Africa at that time, he received a much smaller income than if he had cared for the white community.

**Figure 1.**
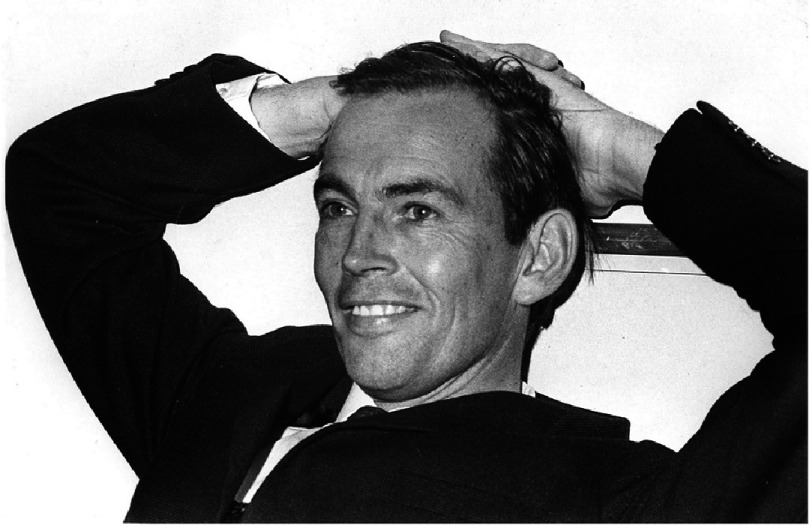
Christiaan Barnard not long after he performed the first heart transplant.

**Figure 2.**
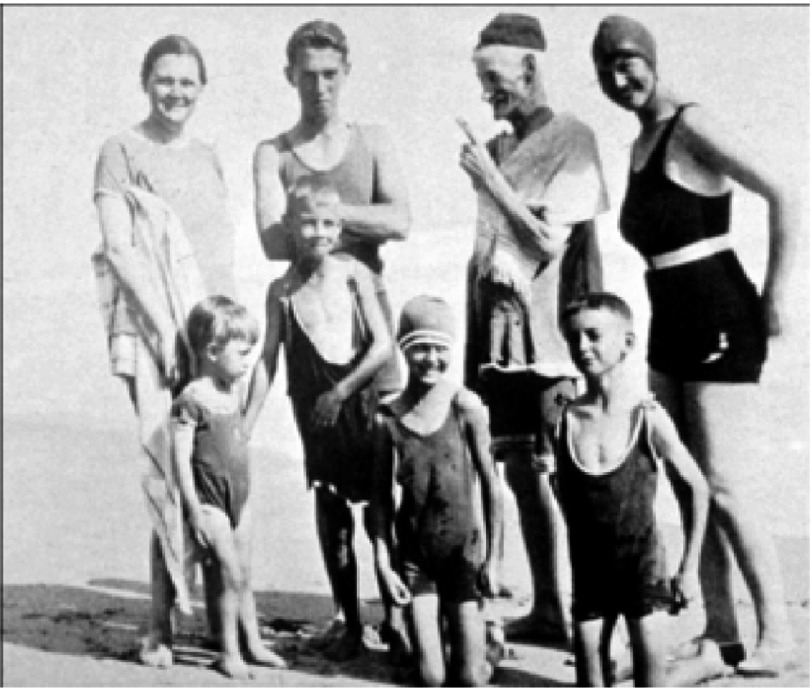
The Barnard family on the beach on vacation when Christiaan was a boy. Back row, left to right: his mother Maria, oldest brother Johannes (Barney), father Adam, and a family friend. Front row: Chris is second from left. The state of their bathing clothes illustrates the family’s relative poverty.

After attending the local high school, Chris Barnard did well enough to gain entry to study medicine at the University of Cape Town (UCT), where he was financially dependent on two scholarships he had been awarded. If he failed an examination, he would lose the scholarships and would no longer be able to pursue his goal of becoming a doctor. He graduated at the end of 1946 and, after house appointments (internships) in Cape Town, he married and, because it promised a steady income, accepted an offer to join a general (primary care) practice in a small town about an hour’s drive inland from Cape Town. He enjoyed this work but, when problems arose between him and his two colleagues, he resigned his position and returned to the Cape Town area to study for higher surgical examinations.

**Figure 3.**
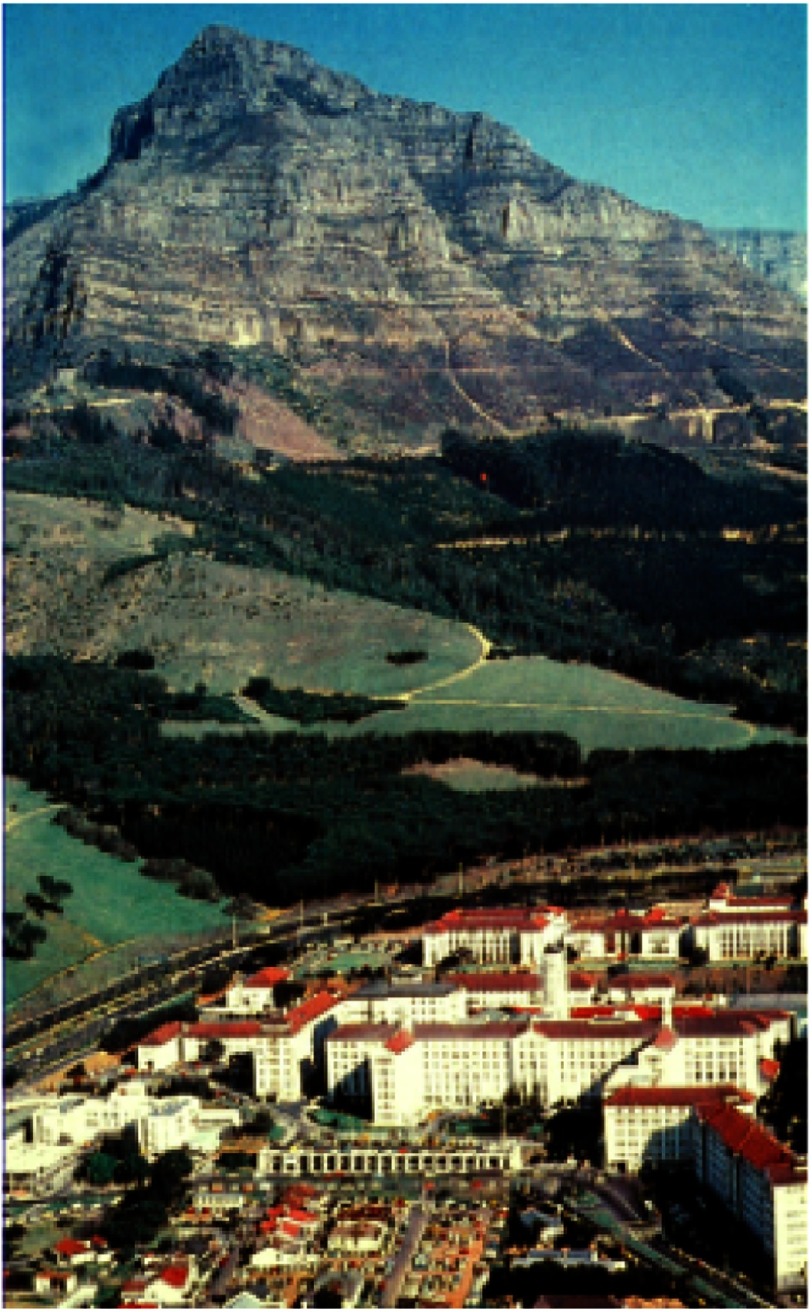
Groote Schuur Hospital, as it was in Chris Barnard’s time. Wildebeest graze on the land behind the hospital. In the 1980s, the small houses in front of the hospital were demolished to allow construction of the present hospital. Fortunately, the beautiful original buildings have been retained, and continue to be used for many purposes.

This proved a difficult period for him as he had no income, but now had two small children to support. Fortunately, a position became available at the local infectious disease hospital and this led to further appointments, first in medicine and then in surgery, at GSH, the major UCT teaching hospital (Figure 3). During this period, in the evenings and nights, he carried out some very innovative research in a canine model of intestinal atresia in neonates^3^.

**Figure 4.**
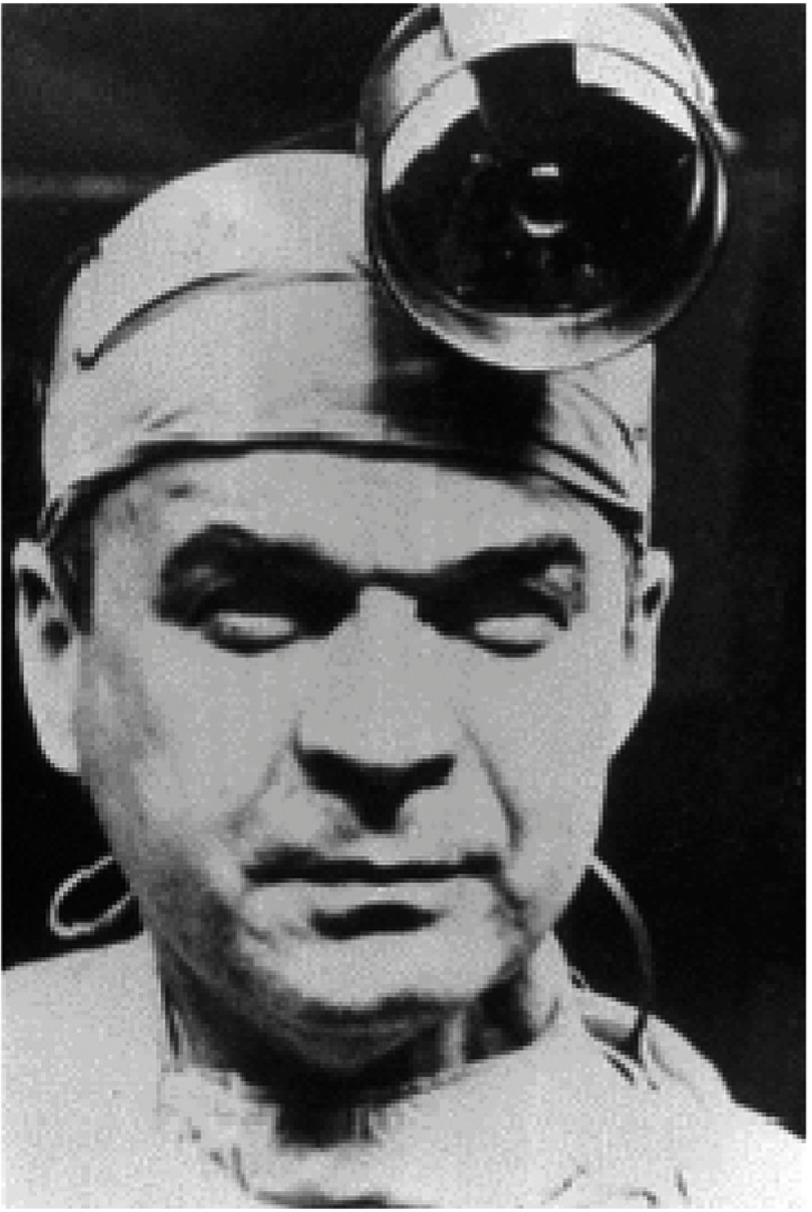
C. Walton (‘Walt’) Lillehei, arguably the greatest pioneer in open heart surgery, and Barnard’s major mentor in Minneapolis.

He was then offered a scholarship to gain surgical experience at the University of Minnesota in Minneapolis under the tutorship of the legendary Professor Owen Wangensteen. It was there that he was first exposed to the very new field of open heart surgery, the University Hospital in Minneapolis being only one of a handful of centers in the world where this form of surgery was being carried out. Barnard immediately saw the potential of the heart-lung machine (pump-oxygenator) and, under the direction of C. Walton (‘Walt’) Lillehei (perhaps the most important of the early open heart surgery pioneers [Figure 4]) and his colleague, Richard Varco, steadily gained experience.

**Figure 5.**
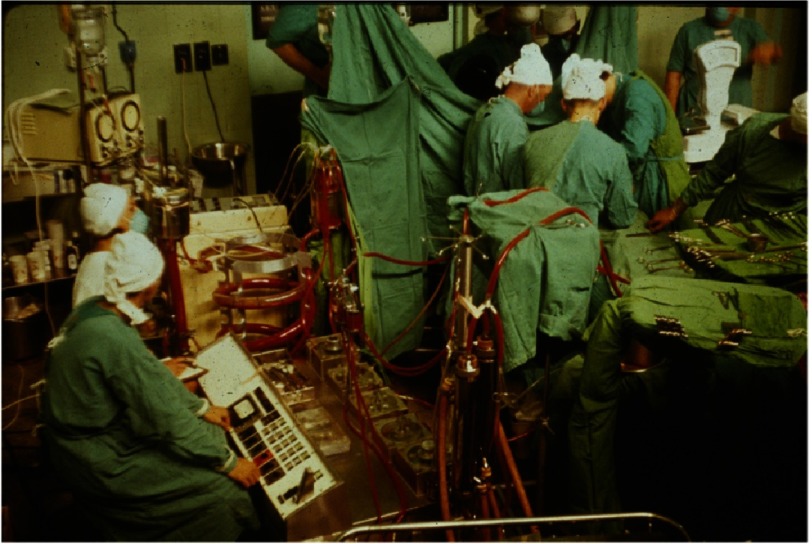
Early open heart operation at Groote Schuur Hospital (1960s). Barnard (with head obscured) is to the right of the operating table.

**Figure 6.**
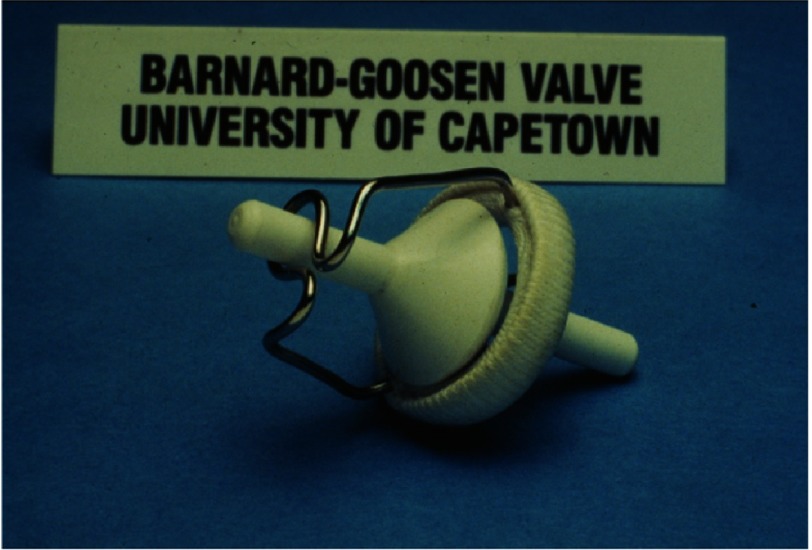
The prosthetic heart valve designed by Barnard and his chief technician, Carl Goosen.

After 30 months in the USA (much of it separated from his family), he returned to UCT with a pump-oxygenator generously provided for him through a grant from the US National Institutes of Health (NIH). He immediately initiated an open heart surgery program at GSH (Figure 5), which was associated with excellent results and gained him a good reputation from those who followed his progress^4–6^. Among his achievements was to become the first surgeon to correct Ebstein’s anomaly^7,8^, and to have probably the world’s best results from the correction of Fallot’s tetralogy^9^. He also designed and implanted a prosthetic valve that was successful for that era (Figure 6)^10–13^.

In the relatively early 1960s, by which time heart surgery could correct most congential heart defects and treat valve disease (but not yet coronary artery disease), he began to consider the future of his specialty. He concluded that heart transplantation would be required if patients with end-stage heart failure were to be helped. (One unsuccessful clinical heart transplant had been attempted by James Hardy in Mississippi using a chimpanzee as a ‘donor’ in 1964^14^.) I personally visited him at GSH in July 1965, where he took me to see a patient who, he had concluded, “needs a new heart”^2^. At the time, I thought perhaps he was joking but, in retrospect, he clearly was not.

## Preparations for the first heart transplant

**Figure 7.**
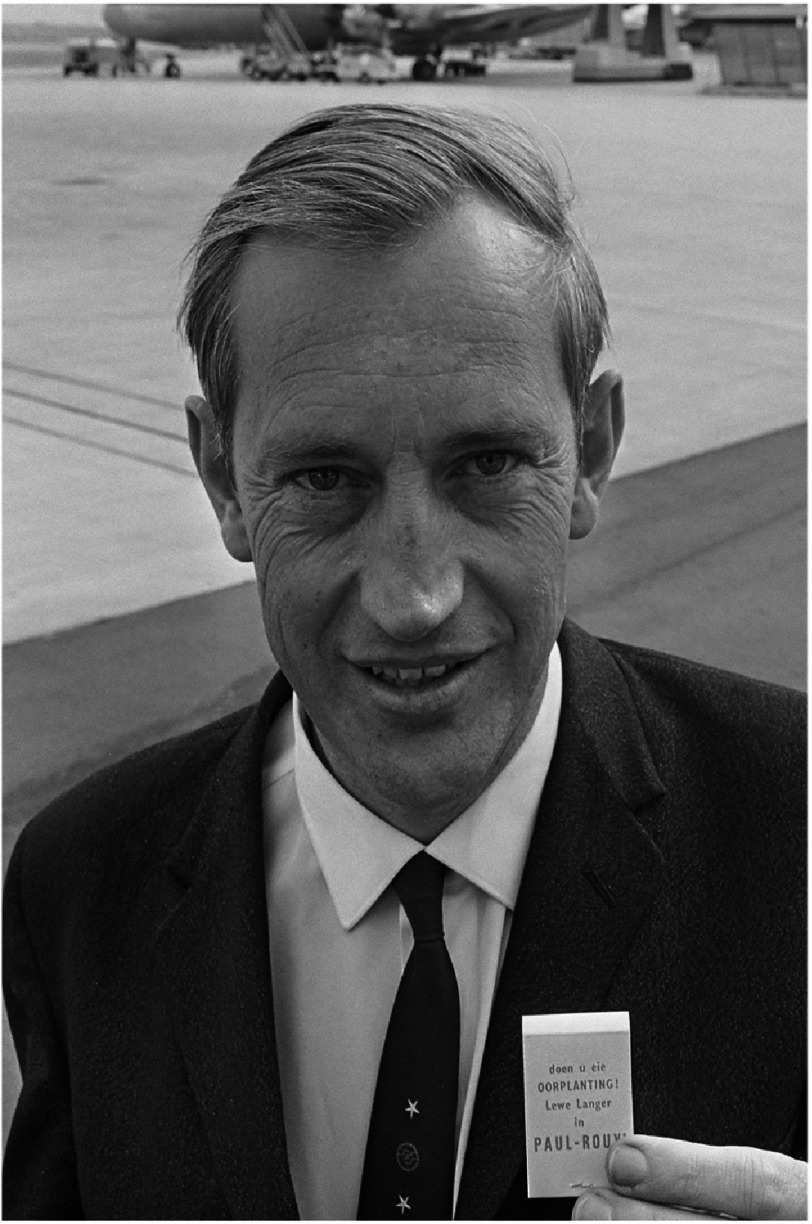
Marius Barnard, younger brother of Chris Barnard.

**Figure 8.**
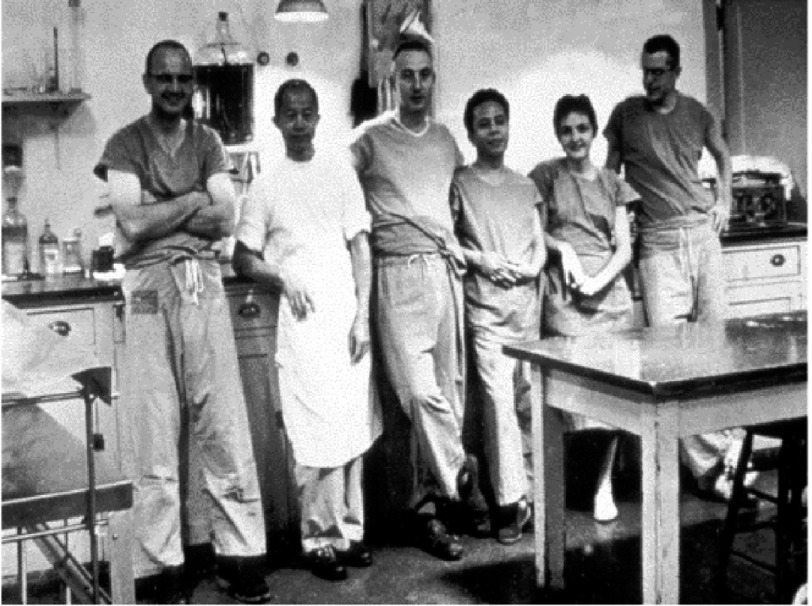
Norman Shumway (third from left) and his research team at Stanford during the early days of his work on experimental heart transplantation. Richard Lower is on the far right.

With a view to moving towards heart transplantation in patients who might benefit from it, Barnard and his younger brother, Marius (Figure 7), who was also a cardiac surgeon at GSH, began by gaining experience of the operation of orthotopic heart transplantation in dogs^15^. He used an operative technique first described by Russell Brock and a junior colleague in London in 1959^16^, but developed and investigated extensively by Norman Shumway and his research team at Stanford University in the USA^17^ (Figure 8). Barnard made little attempt to keep the dogs alive as his main aim was to perfect the surgical technique.

**Figure 9.**
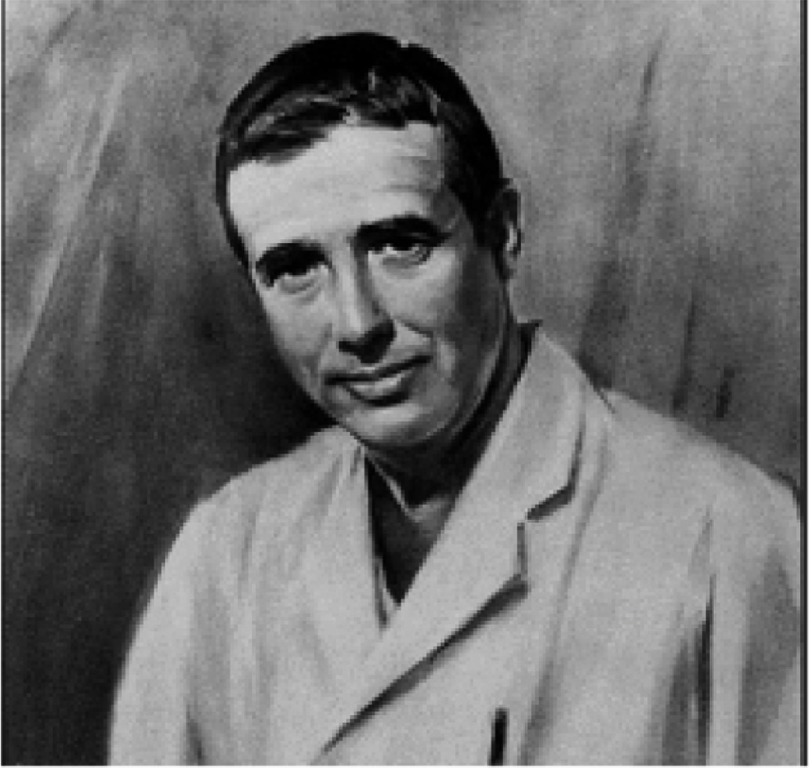
David Hume, from whom Barnard learnt the principles of immunosuppressive therapy in patients with organ grafts.

He then took a three-month sabbatical to gain experience in immunosuppressive therapy in patients with kidney transplants, which he did by attaching himself to the transplant program headed by David Hume (Figure 9) in Richmond, Virginia. There he also gained more experience of experimental heart transplantation in the laboratory of Richard Lower (Figure 8), who had trained with Shumway, but had subsequently been recruited to Richmond by Hume.

With this experience behind him, Barnard returned to GSH and carried out a single successful kidney transplant on a patient who lived for 20 years. Barnard then felt ready to carry out a first heart transplant.

He asked the professor of cardiology, Velva (‘Val’) Schrire, a superb clinician, to select a patient who might benefit from the procedure. In Barnard’s surgical unit in GSH, all patients, regardless of ethnic background, were treated equally, but Professor Schrire believed that selection of a non-white recipient *or* donor might be misinterpreted by the political critics of South Africa as experimenting on the non-white population. They therefore agreed that both recipient and donor should be Caucasian (white).

**Figure 10.**
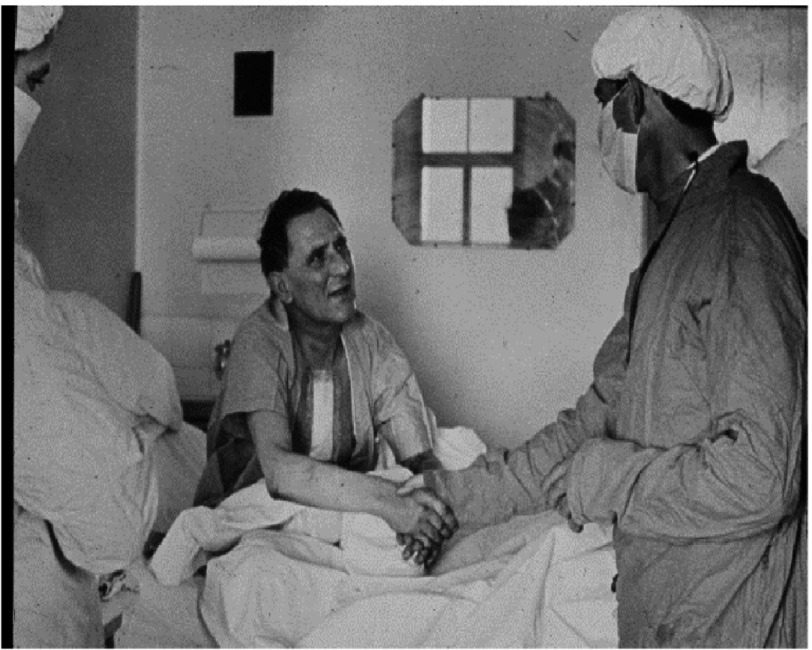
Louis Washkansky as a patient in Groote Schuur Hospital (with Barnard after the heart transplant).

**Figure 11.**
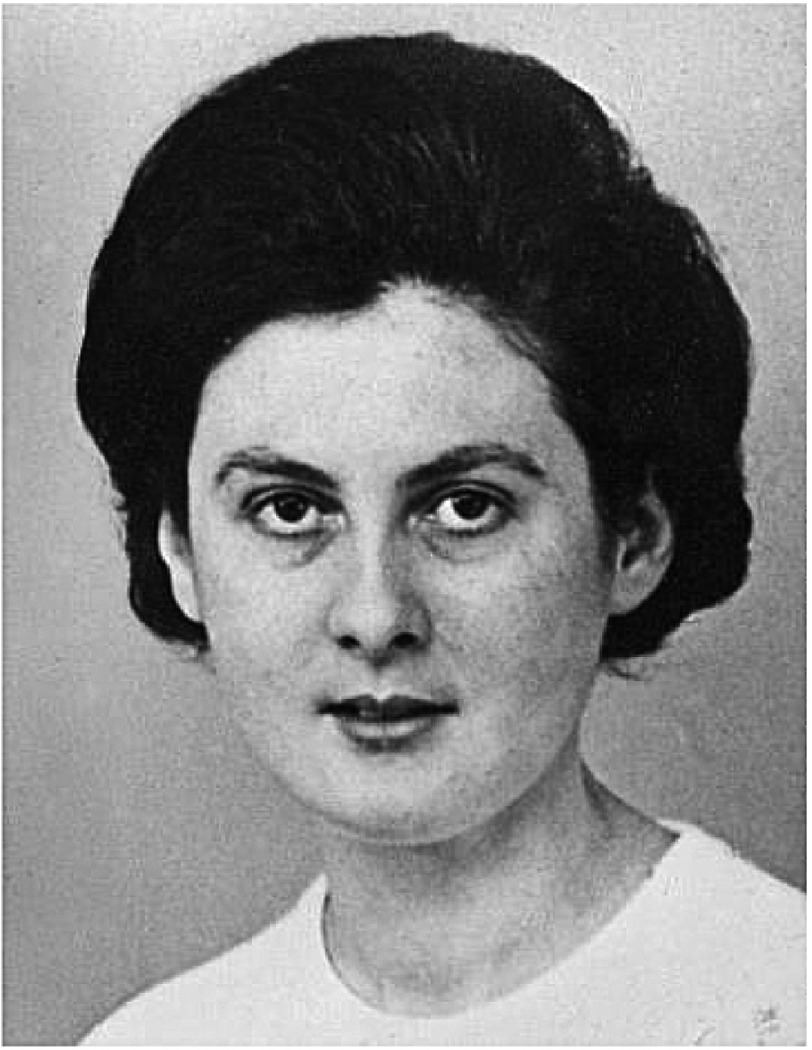
Denise Darvall, the first heart donor.

Schrire identified Louis Waskansky (Figure 10), a 53-year-old diabetic, who was bedridden in hospital in severe cardiac failure from ischemic heart disease^18^. Washkansky readily accepted the opportunity as he knew he had no alternative if he wanted to stay alive. The surgical team then waited for a suitable donor. In the afternoon of Saturday, December 2nd, 1967, Denise Darvall (Figure 11), a 25-year-old woman, was brought to GSH having suffered a severe brain injury as a result of a traffic accident^19^. Within hours, she was certified brain-dead by the hospital neurosurgeons, and her father gave his consent for her heart and kidneys to be used for transplantation.

## The first heart transplant

Both potential recipient and donor were taken to the operating room suite, and the operation took place during the early hours of December 3rd^20^. In that era, the law in South Africa simply stated that a patient was considered dead when he/she was declared dead by a physician. Therefore, Barnard concluded he could use brain death as a criterion for declaring a patient dead. Nevertheless, to be quite sure that he would not be faced by medico-legal problems, he decided he would wait for the heart to stop beating before he removed it. He therefore disconnected the ventilator, and waited until the EKG indicated no cardiac output. This took approximately six minutes.

The chest was then opened quickly by splitting the sternum. The heart was blue and not beating. The surgical team connected the donor to a heart-lung machine, and circulated cold oxygenated blood through her body, with the aim of reducing the metabolism of the heart while it was transplanted. The heart was rapidly cooled to a low temperature, helping to protect it from further ischemic injury during transplantation. The donor heart was excised in such a way that the donor heart-lung machine would continue to perfuse it with cooled oxygenated blood while it was carried into the adjacent recipient operating room. Thus, the heart continued to be protected from injury.

This approach is rarely followed today when a beating donor heart is simply cooled to a very low temperature by perfusing it with a cold preservation solution, then excised, and covered in ice or cold saline. However, the continuous perfusion of the donor heart with oxygenated blood in Mr. Washkansky’s case may have been important as Barnard had allowed the heart to suffer an insult and possible injury while it stopped beating, which is much less commonly allowed today.

Although Barnard and two of his colleagues, including his brother, Marius, had carried out a relatively large number of heart transplants in dogs, Chris’s two major assistants on the day of this first clinical operation *had never seen a heart transplant in their lives before – not even in a dog.* I found it quite remarkable that the team had not practiced the operation together.

**Figure 12.**
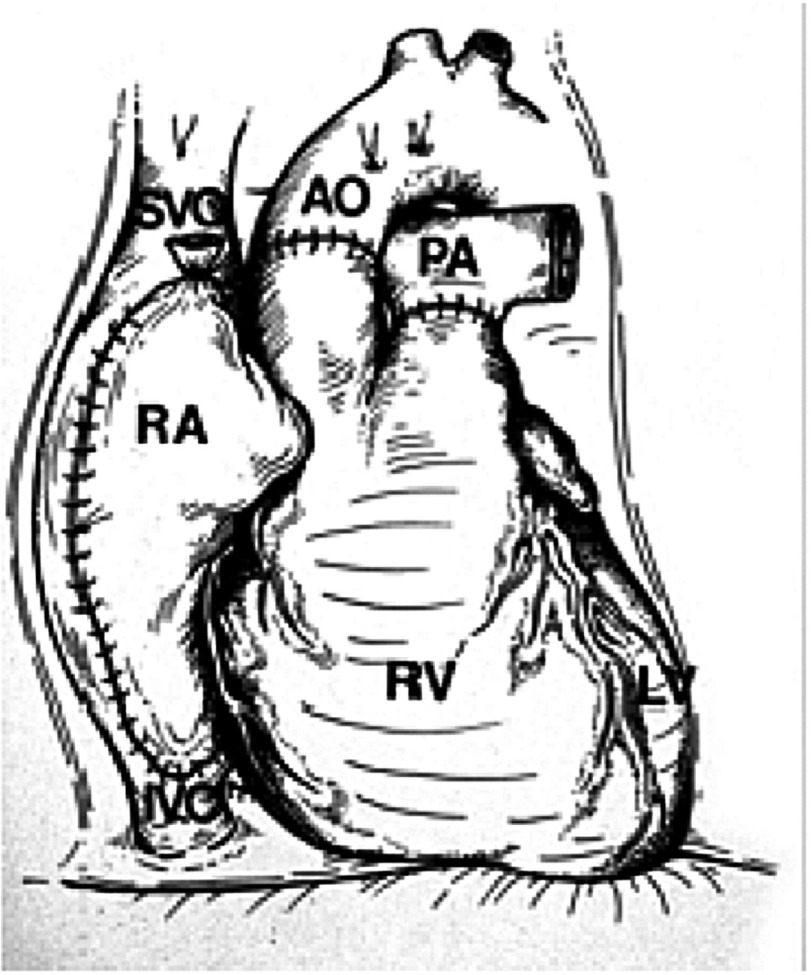
The operation of orthotopic heart transplantation, in which both native ventricles and all four cardiac valves are excised, and anastomoses are made between the donor and recipient left atria (not shown), the two right atria (RA), the aortae (AO), and pulmonary arteries (PA). (LV, left ventricle; IVC, native inferior vena cava; RV, right ventricle; SVC, native superior vena cava).

“Louis Washkansky’s heart was then removed,” Chris told me, “and, for the first time in my life, I stared into an empty chest.” (Although by this stage of his career, he had carried out hundreds, if not thousands, of heart operations, he had never before looked into a human chest and seen *no* heart.) “At that moment, the full impact of what I was attempting became abundantly clear to me.” The donor heart was quickly sewn in place without difficulty (Figure 12).

Over the previous few years, Mr. Washkansky’s diseased heart had become dilated when it failed to cope with the blood it was struggling to pump around his body. It was therefore much larger than a normal healthy heart. Denise Darvall’s heart was much smaller than even a healthy man’s heart, and so it looked tiny in the large space left by Mr. Washkansky’s heart. Barnard looked at it and wondered whether it was too small to support the circulation in such a relatively big man. This observation must have been very disturbing to him, but he could do nothing about it now.

Once Barnard had completed the transplant, he allowed the blood from the recipient’s heart-lung machine to perfuse through the new heart. By warming the blood as it passed through the heart-lung machine, he also raised the patient’s body temperature back to normal. The surgical team waited for the heart to beat, but for some minutes it refused to do so (although it was fibrillating). Barnard became increasingly worried that the heart muscle had been severely damaged when he had disconnected the donor’s oxygen supply. He electrically defibrillated the heart, and at last it began to contract normally, but only weakly, and would not take over the circulation.

Barnard tried twice to wean the patient from pump-oxygenator support, but the heart was not beating strongly enough to maintain an adequate blood pressure. He allowed more time for the donor heart to gain strength, continuing to keep the patient alive on the heart-lung machine. Steadily the beats became stronger. At the third attempt (to discontinue the heart-lung machine), the blood pressure kept rising. “Naturally, I felt a great sense of relief,” Barnard told me. The heart-lung machine could now be switched off, and the chest closed. The operation had been successful. From ‘skin to skin’, it had taken almost 5 h. It was 6.15 a.m. Chris reached across the operating table and shook his chief assistant’s gloved hand.

When he was satisfied the heart was beating well and the patient would recover, Chris left his colleagues to close Mr. Washkansky’s chest.

## The immediate post-operative period

Barnard had not informed either the hospital’s medical superintendent nor the chairman of the department of surgery that he was about to carry out this historic operation, but he now decided he should do so. The hospital superintendent, Dr Jacobus Burger, was surprised to learn of the operation, but pleased that the patient was doing well. The chairman of surgery was equally pleased, but wondered why Barnard had not been in touch with him before he began the operation. “I didn’t think it was necessary,” replied Barnard, suggesting that he did not realize the impact the operation would make worldwide.

However, he must have had some realization that the operation he had just performed was special because he also telephoned an old friend from their medical student days who was a member of the Executive Committee in charge of Hospital and Health Affairs in the Cape Provincial Administration, a political position of some influence^21^. This politician immediately recognized the significance of the transplant, and informed the Administrator of the Cape Province (similar to a State Governor in the USA) who, in turn, telephoned the Prime Minister of South Africa.

The importance of this unique operation to South Africa is evident by the fact that, within about 30 minutes of Barnard leaving the operating room, the Prime Minister of the country had been informed. The politicians had immediately realized its potential impact on the world. It could put South Africa on the medical map. Because of the poor international reputation resulting from the government’s official apartheid policy, the operation was more important for South Africa than it might have been for most other countries. In contrast, from my many discussions with Barnard, I do not think he had any idea of the impact the operation would make on South Africa’s standing in the world.

“There were no photographers at the first transplant,” Chris told me, “not because we wanted to keep them away, but because we honestly didn’t think it was a big deal.”

How wrong could he be? Within hours, the hospital received an offer of US$1 million for a photograph of the donor heart being placed into the chest of Mr. Washkansky on the operating table, but, of course, no photographs had been taken. Furthermore, when he left the operating room, Barnard discarded his surgical gloves in the trash can as usual, only to learn a few days later that a newspaper was offering to purchase them for US$25,000.

**Figure 13.**
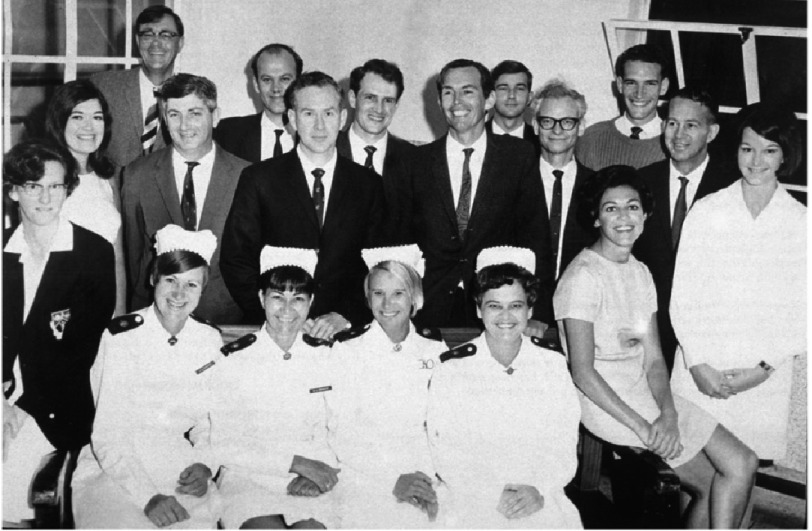
The first photograph of the surgical team taken on the afternoon of the day of the first heart transplant.

## The media onslaught begins

When he had seen Mr. Washkansky settled safely in his intensive care room, Barnard drove home. It was only an hour later when phone calls came from all over the world. Chris told me many times that he and his colleagues were stunned by the interest the transplant engendered. So great was the public interest that the entire team was called back into the hospital that afternoon to be photographed (Figure 13).

**Figure 14.**
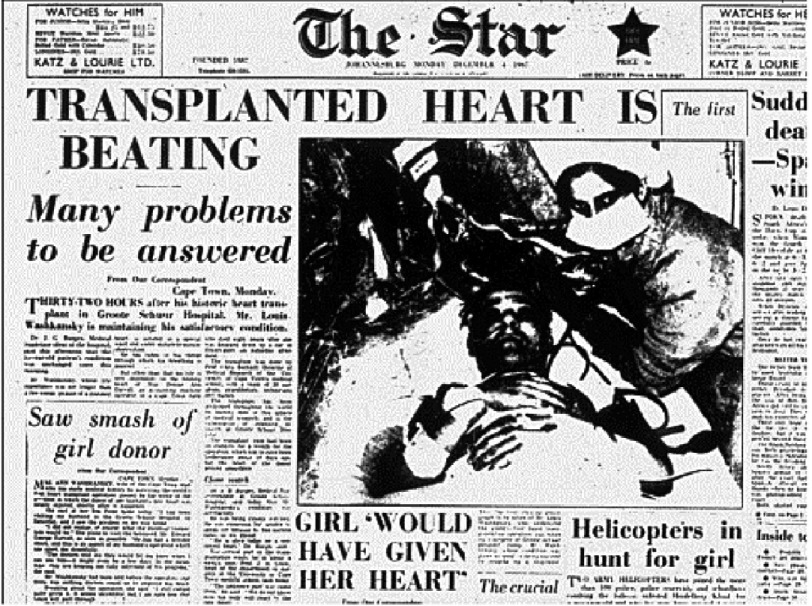
The front page of a South African newspaper on the day following the first heart transplant.

It may perhaps be difficult for some to appreciate what is meant by ‘media attention’. Journalists and photographers flew in from all over the world and swarmed over Groote Schuur Hospital. Radio and television services worldwide provided bulletins and updates on Mr. Washkansky’s progress throughout the day. On the front page of several local newspapers, a photo showed Louis Washkansky, and was transmitted around the world within twenty-four hours (Figure 14). Indeed, the whole world watched the patient’s progress.

## Mr. Washkansky’s progress

“Mr. Washkansky’s immediate recovery was excellent,” recalled Barnard. “For the first time in medical history, we were able to observe the effect that a healthy transplanted heart had in a patient who, until that time, was in severe heart failure.

After the first week, Mr. Washkansky began to feel tired and less well. In retrospect, it is clear that his recovery was impaired by allowing him to have too many visitors and give too many interviews to the media. After approximately 12 days, his condition began to deteriorate, and he developed radiographic infiltrates in the lungs. These were erroneously believed to be a result of a condition David Hume had described as “transplant lung”, a reaction in the lungs in response to rejection in the transplanted organ (that was later disproved). Influenced from what he had learnt in Richmond, Barnard unfortunately increased the patient’s immunosuppressive therapy until it became clear that the patient had pneumonia. Despite intensive antibiotic treatment, Mr. Washkansky deteriorated rapidly and died in the early hours of Thursday, December 21st, 18 days after the transplant. Ironically, Denise Darval’s heart was the last organ to fail. Exhausted by his efforts to keep his patient alive, Barnard was devastated by this sad outcome.

An autopsy was carried out immediately by the professor of pathology, James Thompson^22^. He could find no features of rejection of the heart, and confirmed that death had resulted from pneumonia. By inspecting the suture lines, he ascertained that Barnard had performed the operation faultlessly.

**Figure 15.**
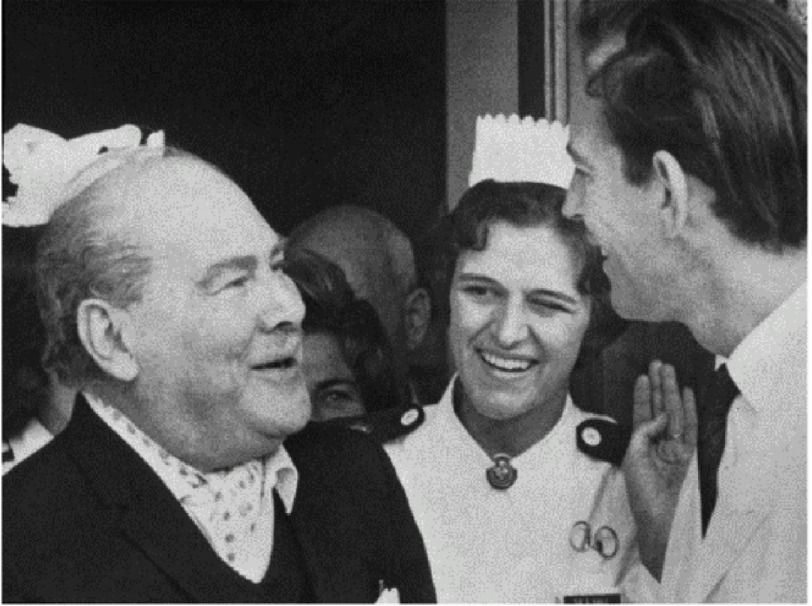
Philip Blaiberg leaving Groote Schuur Hospital after his heart transplant.

## The second heart transplant

Barnard bounced back from his disappointment over the outcome of Mr. Washkansky’s operation, and soon added a second patient to the waiting list for a donor heart. The patient was a retired dental surgeon, Philip Blaiberg (Figure 15), who was in a similar clinical state of terminal cardiac failure as Mr. Washkansky had been. A donor became available on January 2nd, 1968, and the operation proceeded satisfactorily. Dr Blaiburg was discharged from GSH on the 74th post-operative day, and lived a fairly full and active life for almost 19 months^23^, eventually dying from the hitherto unknown condition of graft atherosclerosis (chronic rejection)^24^.

Barnard’s third patient, Petrus Smith, lived for 20 months, a month longer than Dr. Blaiberg. Even though Mr. Washkansky had survived for only 18 days, Barnard’s first four patients lived an average of more than 200 days, in great contrast to the majority of other patients operated on at other centers worldwide, who, even if they survived the operation, often died within a few days or weeks. Remarkably, his fifth patient lived for almost 13 years, and his sixth for more than 23 years^25–27^.

**Figure 16.**
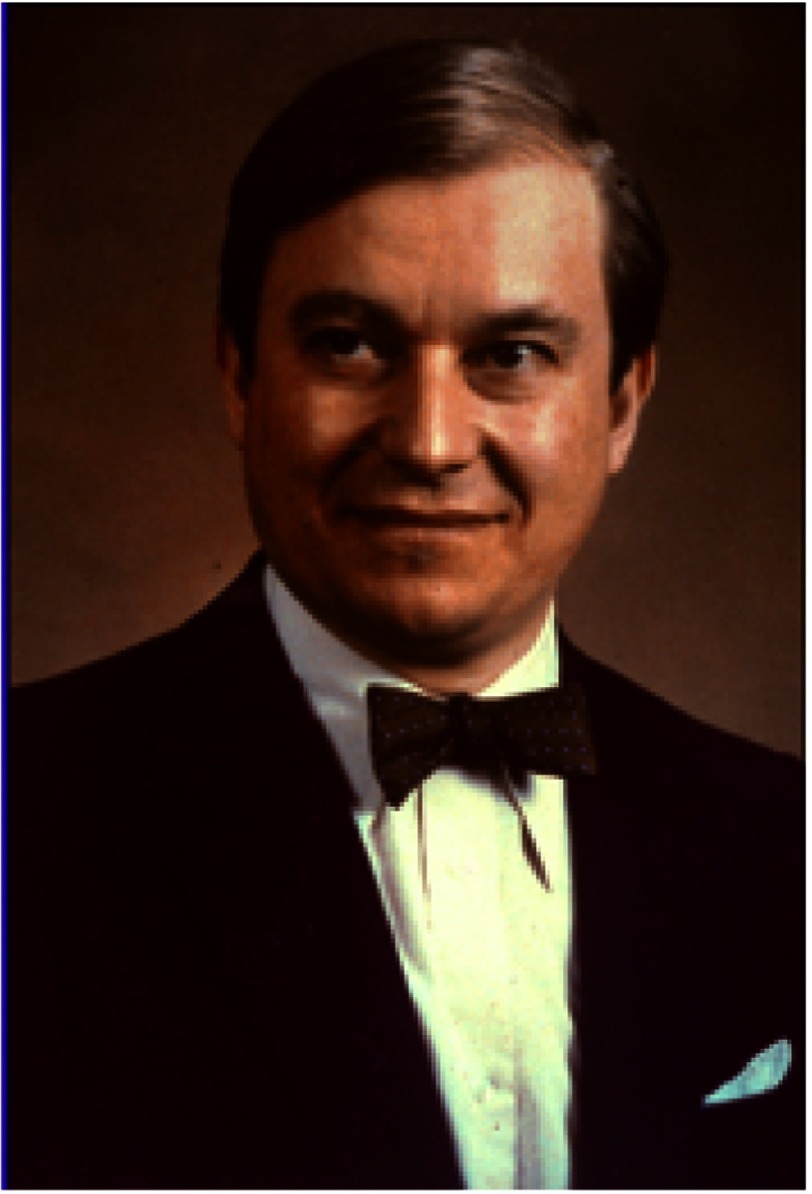
Belgian surgeon, Jacques Losman, who, with Barnard, developed the operation of heterotopic heart transplantation.

**Figure 17.**
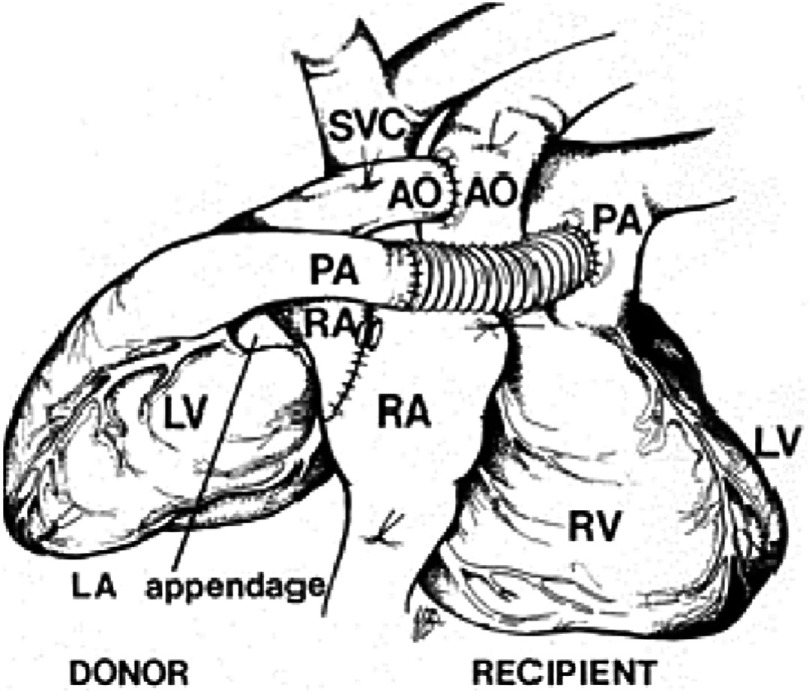
The operation of heterotopic heart transplantation, in which both the native left and right ventricles are provided with assistance by the donor heart (which is placed in the right side of the chest). AO, aorta; LA, left atrium; LV, left ventricle; PA, pulmonary artery; RA, right atrium; RV, right ventricle; SVC, superior vena cava.

## Subsequent career

Unfortunately, the immense media attention directed towards the attractive and articulate Barnard steadily distracted him from his work, as he was seemingly always willing to accept any invitation to speak anywhere in the world. The heart surgery program at GSH was sustained by his colleagues, increasingly led by his very competent younger brother, Marius, but Barnard’s day-to-day leadership and vision were obviously missed.

Nevertheless, after experiencing some early graft failures from ischemia-reperfusion injury, in 1975 he set his junior colleague, Jacques Losman (Figure 16), to design an operation in which the transplant was inserted as an auxiliary heart, in a heterotopic position (Figure 17)^28,29^. This enabled some help from the patient’s native heart (with inotropic support) in the event the donor heart functioned poorly in the immediate post-transplant period, and also when it was suffering from a severe rejection episode^30,31^. Heterotopic heart transplantation played a significant role in the GSH program for several years but, when thyroid hormone therapy to the donor was introduced (resulting in improved immediate post-transplant function), and when the severity of rejection episodes was minimized by the new immunosuppressive agent, cyclosporine^32,33^, Barnard’s unit reverted to orthotopic heart transplantation in the majority of cases.

**Figure 18.**
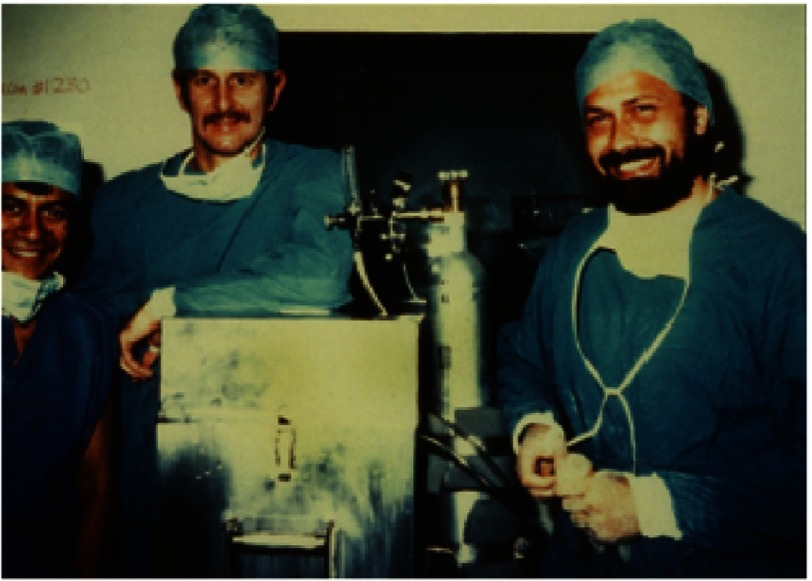
Winston Wicomb (left) and Dimitri Novitzky (right), with the author, on the occasion of the world’s first use of a hypothermic perfusion storage system to transport a human donor heart (in 1981).

Before Barnard’s retirement, his junior research colleague, Winston Wicomb (Figure 18), had introduced hypothermic perfusion of the donor heart as a means of protecting the heart during transportation to GSH^34,35^, enabling for the first time hearts to be procured at centers far distant from Cape Town. In addition, Barnard’s junior surgical colleague, Dimitri Novitzky (Figure 18), was carrying out his groundbreaking studies on the pathophysiology of brain death and the beneficial effects of thyroid hormone therapy on donor heart function^36–38^, a therapy that is now used worldwide.

## Retirement

**Figure 19.**
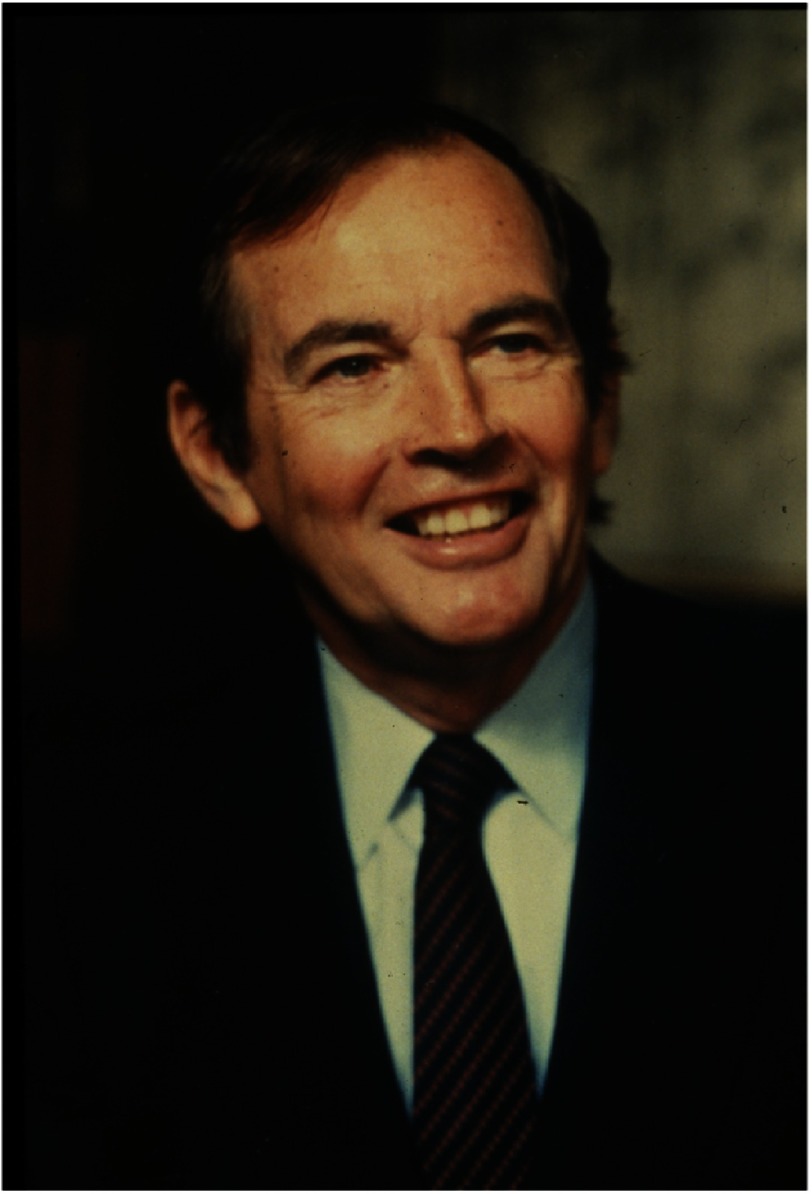
Christiaan Barnard at about the time of his retirement from Groote Schuur Hospital at the end of 1983.

As the years passed, Barnard steadily lost his drive and passion for heart surgery and, at the age of 61, he took early retirement from GSH at the end of 1983 (Figure 19). He was then able to spend more time on the many other interests he had developed since the first heart transplant. These included various business ventures, public speaking (in which he was outstanding), and writing^2,39^. For eight years he contributed a weekly column for a Cape Town newspaper that was syndicated throughout South Africa, and he wrote or edited several books on various aspects of health care for the lay public. In addition, sometimes with professional help, he wrote two autobiographies^1,40^, four novels, and a book in which he discussed South Africa’s political problems and his suggestions for resolving them^41^.

In 1986, he accepted an invitation to help surgeons in Oklahoma City establish a heart transplantation program. Although he did not participate in the actual surgery or care of the patients, his advice was valuable and, through his public relations activities, he did much to establish a successful program.

**Figure 20.**
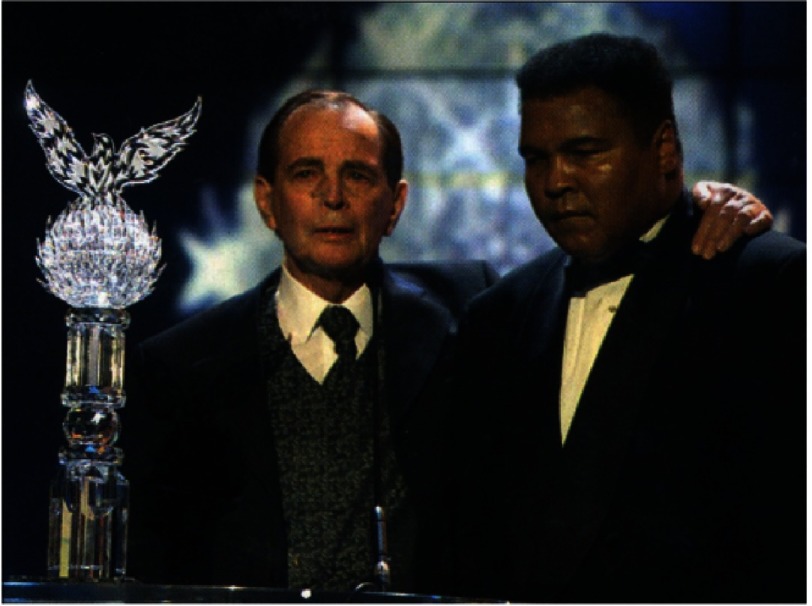
Christiaan Barnard, his face scarred by treatment for a basal cell carcinoma, was asked to present the sports personality of the century award to the American former boxer, Muhammad Ali. To me, this photograph of two of the 20th century’s most iconic men, by then well past their prime, is one of the saddest I have ever seen.

His personal life was chequered^2^. He was married and divorced three time, having two children with each wife. Complicated by ill health and loneliness, his last few months were the most miserable of his life (Figure 20). He died suddenly on September 2nd, 2001, aged 78.

## Barnard’s legacy

How will Christiaan Barnard be remembered? What will be his legacy?

He will be remembered as the surgeon who had the courage to carry out the world’s first heart transplant using a heart from a deceased human donor. Today, when heart transplantation has become a relatively routine and commonplace procedure, we may be inclined to underestimate Barnard’s immense confidence and courage in undertaking this first operation. By any standard, it was a monumental step to take. In particular, his courage (and some might say ‘recklessness’) in waiting while the donor heart was allowed to cease beating (to ‘die’) before it was excised, is exceptional, as this could have resulted in primary failure of the graft to support the patient’s circulation. Maybe the few other surgeons who were planning heart transplants at the time did not have the same courage, particularly those in countries where litigation against doctors was more common. Furthermore, Barnard’s introduction of heterotopic heart transplantation was innovative and played a role in those early days when the results of orthotopic heart transplantation were poor.

However, he will also be remembered as a man who enjoyed life to the full, and employed his personality, sense of humor, and articulacy to inform and entertain thousands, if not millions, through his public lectures and appearances on television. Unfortunately, his personal life and public behavior at times distracted from his role as a leading member of the medical profession, and he lost the support of some of his colleagues, who perceived him as something of a ‘playboy’. Nevertheless, these activities should not detract from the significant contributions he made to advancing heart surgery.

To me personally, Chris Barnard was simply the most interesting, stimulating, and charismatic person I have had the good fortune to know in my relatively long life – an unforgettable man.

“It is not the critic who counts; not the man who points out how the strong man stumbled, or whether the doer of deeds could have done them better. The credit belongs to the man who is actually in the arena, whose face is marred by dust and sweat and blood; who strives valiantly, who errs and comes short again and again; who knows the great enthusiasms, the great devotions; who spends himself in a worthy cause; who, at the best, knows in the end the triumph of high achievement, and who, at the worst, if he fails, at least fails while daring greatly, so that his place shall never be with those timid souls who know neither victory nor defeat.”

Theodore Roosevelt

David Cooper’s biography of Christiaan Barnard was published by Fonthill Media in November 2017.
